# Correction to: Drinking water contamination from perfluoroalkyl substances (PFAS): an ecological mortality study in the Veneto Region, Italy

**DOI:** 10.1093/eurpub/ckad147

**Published:** 2023-08-14

This is a correction to: Marina Mastrantonio and others, Drinking water contamination from perfluoroalkyl substances (PFAS): an ecological mortality study in the Veneto Region, Italy, *European Journal of Public Health*, Volume 28, Issue 1, February 2018, Pages 180–185, https://doi.org/10.1093/eurpub/ckx066

In the originally published online version of this manuscript, there were errors in the final figures of Table 1, with duplication of figures in last 5 values of the Contaminated area column SMRate with values in column SE.

Table 1 should read:

**Table 1:** Number of deaths (Deaths), standard mortality rates (SMRate) and relative standard errors (SE) for PFAS contaminated and uncontaminated municipality areas in males in the period 1980–2013. Relative risk (RR) and 95% confidence intervals are also reported. Statistically significant RR are evidenced in bold

Mortality causes	Contaminated area			Uncontaminated area			
	Deaths	SMRate	SE	Deaths	SMRate	SE	RR
All causes	21149	1107.53	7.63	73 745	1007.45	3.72	**1,10** (1.08-1.10)
Liver cancer	242	13.68	0.88	1132	15.31	0.46	0.89 (0.78–1.03)
Kidney cancer	155	8.69	0.70	602	8.09	0.33	1.07 (0.90–1.28)
Bladder cancer	225	12.96	0.87	830	11.54	0.40	1.12 (0.97–1.30)
Pancreatic cancer	361	20.55	1.08	1355	18.48	0.50	1.11 (0.99–1.25)
Leukemia	210	11.71	0.81	749	10.11	0.37	1.16 (0.99–1.35)
Non-Hodgkin’s lymphoma	155	8.81	0.71	610	8.24	0.34	1.07 (0.90–1.28)
Breast cancer	4	0.22	0.11	18	0.24	0.06	0.89 (0.30–2.65)
Prostate cancer	401	23.46	1.17	1654	23.47	0.58	1.00 (0.90–1.12)
Testicular cancer	8	0.44	0.16	19	0.24	0.05	1.86 (0.81–4.27)
Diabetes	292	16.74	0.98	1002	13.83	0.44	**1.21** (1.06-1.38)
Cerebrovascular disease	1871	108.45	2.51	5776	80.97	1.07	**1.34** (1.27-1.41)
Myocardial infarction	1900	107.91	2.48	6478	88.43	1.10	**1.22** (1.16-1.28)
Alzheimer’s disease	89	5.22	0.55	274	3.91	0.24	**1.33** (1.05-1.70)
Parkinson’s disease	88	5.18	0.55	307	4.39	0.25	1.18 (0.93–1.50)

instead of:

**Table 1:** Number of deaths (Deaths), standard mortality rates (SMRate) and relative standard errors (SE) for PFAS contaminated and uncontaminated municipality areas in males in the period 1980–2013. Relative risk (RR) and 95% confidence intervals are also reported. Statistically significant RR are evidenced in bold

Mortality causes	Contaminated area			Uncontaminated area			
	Deaths	SMRate	SE	Deaths	SMRate	SE	RR
All causes	21149	1201.57	8.28	73 745	1007.45	3.72	**1.19** (1.17–1.21)
Liver cancer	242	16.68	0.88	1132	15.31	0.46	0.89 (0.78–1.03)
Kidney cancer	155	8.69	0.70	602	8.09	0.33	1.07 (0.90–1.28)
Bladder cancer	225	12.96	0.87	830	11.54	0.40	1.12 (0.97–1.30)
Pancreatic cancer	361	20.55	1.08	1355	18.48	0.50	1.11 (0.99–1.25)
Leukemia	210	11.71	0.81	749	10.11	0.37	1.16 (0.99–1.35)
Non-Hodgkin s lymphoma	155	8.81	0.71	610	8.24	0.34	1.07 (0.90–1.28)
Breast cancer	4	0.22	0.11	18	0.24	0.06	0.89 (0.30–2.65)
Prostate cancer	401	23.46	1.17	1654	23.47	0.58	1.00 (0.90–1.12)
Testicular cancer	8	0.44	0.16	19	0.24	0.05	1.86 (0.81–4.27)
Diabetes	292	0.98	0.98	1002	13.83	0.44	**1.21** (1.06–1.38)
Cerebrovascular disease	1871	2.51	2.51	5776	80.97	1.07	**1.34** (1.27–1.41)
Myocardial infarction	1900	2.48	2.48	6478	88.43	1.10	**1.22** (1.16–1.28)
Alzheimer s disease	89	0.55	0.55	274	3.91	0.24	**1.33** (1.05–1.70)
Parkinson s disease	88	0.55	0.55	307	4.39	0.25	1.18 (0.93–1.50)

This error has been outlined only in this correction notice to preserve the version of record

